# Smoking differences between employees in faculties of the University of Tartu, Estonia, and changes during the country's transition

**DOI:** 10.1186/1471-2458-11-153

**Published:** 2011-03-08

**Authors:** Simo Näyhä, Jana Kivastik, Peet-Henn Kingisepp, Rauno Heikkinen

**Affiliations:** 1 Institute of Health Sciences, University of Oulu, P. O. B. 5000, FI-90014 University of Oulu, Finland; 2 Finnish Institute of Occupational Health, Aapistie 1, FI-90220 Oulu, Finland; 3 Department of Physiology, University of Tartu, Ravila 19, Tartu 50411, Estonia; 4 Institute of Biomedicine, University of Oulu, P. O. B. 5000., FI-90014 University of Oulu, Finland

## Abstract

**Background:**

A previous study found marked differences in smoking between employees in various university faculties in Tartu, Estonia, soon after the disruption of communism. The present study was conducted to see whether such differences still exist and how the patterns had changed during the country's first transitional decade.

**Methods:**

All employees at the University of Tartu (UT) were surveyed for smoking habits by means of a questionnaire in 1992 and 2003. The present paper is based on respondents whose faculty or workplace was known (1390 people in 1992, 1790 in 2003). Smoking differences were assessed in terms of regression-based adjusted figures.

**Results:**

While 20% of the male employees smoked daily in 1992, 13% did so in 2003, the figures for females being 10% and 7%, respectively. The prevalence of men's daily smoking varied between faculties and other workplaces in the range 4-30% in 1992, and 0-24% in 2003, with corresponding ranges of 3-21% and 0-10% among females. Men in the medical faculty in both surveys, and those in the faculty of philosophy in the second survey showed higher rates than men in most other faculties, as did women in the faculty of law in the first survey and those in the faculty of philosophy in the second. The figures were usually low in the faculties of sports & exercise, physics & chemistry and mathematics. The sex pattern was reversed in the faculty of law and also in that of economics, where the women smoked more than the men.

**Conclusions:**

Even in this low-smoking academic community, wide smoking differences existed between the faculties and other workplaces. Faculties where physical or mental performance is of prime importance are leading the way towards a smoke-free community, while men in the faculty of philosophy and, paradoxically, men in the medical faculty are lagging behind. The reversed sex ratio in the faculties of law and economics may indicate women's intensified drive for equality in this transitional society. We assume that different professional cultures may introduce variations in smoking patterns, thereby modifying the course of the smoking epidemic.

## Background

When low smoking rates were observed among doctors in the 1960's they were attributed not only to better awareness of the hazards of smoking within the medical profession but also to an unidentified factor related to academic education. Thus Lynch [1], for example, found low smoking rates among employees in both medical and non-medical faculties, and Brown and Gunn [2] reported low rates among employees in a university where no medical faculty existed. The low prevalence of smoking among the educated people is well-known nowadays [3,4], but few studies have looked for variations within educated communities such as universities. It would seem likely that the scientific career pursued in a university, or the particular academic culture in which one works, could affect smoking habits through social learning. We conducted a smoking survey in the University of Tartu (UT), Estonia, soon after the disruption of communism (1992) and found low but still widely varying smoking rates among the staff and students in different faculties, the prevalence of daily smoking ranging from zero to 30% [5]. Smoking was most common in faculties having the closest connection with the contemporary changes in society, such as the faculty of theology, and among female employees in the faculties of law and economics, while the rates were very low in the faculties of exercise & sports and mathematics, where physical or mental performance are of particular significance.

Since Estonia's transition to a western economy may have modified smoking patterns, the UT employees were re-surveyed in 2003, two years before the enforcement of the Estonian Tobacco Act. In general, we expected to see an overall decline in smoking rates throughout the faculties-despite persistently high national rates. This could be predicted from innovation diffusion theory, which presupposes that new trends in smoking are first adopted by the most educated people [6]. In particular, we tested the hypothesis that smoking differences between the faculties still exist after the first transitional decade and are similar to those observed immediately after the disruption of communism. Any departures from the previous pattern would suggest that the changes in Estonian society during the 1990's had interfered with smoking trends in different ways depending on the faculty. Information on smoking trends among the highest educated professionals would also add to our understanding of the natural course of the smoking epidemic in transitional societies.

## Methods

### Data sources

In the first smoking survey, carried out among all the employees of the University of Tartu (UT), Estonia, between autumn 1992 and spring 1993, a questionnaire was sent to 1930 people, of whom 1441 (75%) returned it. The survey was repeated between December 2003 and April 2004, a questionnaire being sent to all 2691 employees, of whom 2117 (79%) replied. Each respondent was asked to indicate the faculty or institution in which he or she currently worked. The eight faculties existing in 1992 and the ten in 2003 were all included, together with the administrative section and the library (1390 and 1790 persons in 1992 and 2003, respectively). Separate institutions and the five out-of-faculty colleges were excluded, as was the faculty of theology, where numbers were too small. The study was approved by the Ethics Review Committee on Human Research of the University of Tartu.

### Description of participants

The average age of the respondents was 43 years (range 18-80) in 1992 and 44 years (17-83) in 2003. Seven percent were over 65 years of age. The characteristics of the sample are summarized by faculties and other workplaces in Table 1. The response rate ranged from 60% to 92% in the various faculties, exceeding 70% in most of them. The proportion of women increased between the surveys, with the greatest increase in the faculties of law (from 45% to 76%) and economics (from 47% to 65%). The majority of respondents in most faculties belonged to the higher occupational group, but almost all those working in the administrative section and library belonged to the lower group. In the 2003 survey, which also asked about education, 98% of the respondents in the higher occupational group and 49% in the lower group had an academic degree (bachelor, master or doctor).

**Table 1:** Description of the participants in the two successive surveys-numbers of respondents (No.), response rate, percentage of men, mean age and standard deviation (SD), and percentage belonging to the higher occupational group

Faculty/workplace	SURVEY I (1992)						SURVEY II (2003)					
		
	**No**.	Response rate (%)	Men (%)	Age (yr)		Higher occupational group^†^(%)	**No**.	Response rate(%)	Men (%)	Age (yr)		Higher occupational group^† ^(%)
										
				Mean	SD					Mean	SD	
Administration	111	79	24	44	14	8	268	92	46	47	15	0
Biology & Geography	78	74	58	39	12	58	176	75	46	41	13	61
Economics & Business	55	81	53	42	13	69	66	83	35	45	14	65
Education	-	-	-	-	-	-	106	84	20	41	12	44
Exercise & Sports Science	55	86	47	45	12	72	48	80	40	42	13	71
Law	56	90	55	44	16	66	29	60	24	43	15	59
Library	164	77	11	40	13	1	192	82	14	46	13	2
Medicine	335	73	42	45	14	68	363	82	29	44	12	62
Mathematics & Computing	114	74	55	44	13	54	68	73	49	45	14	67
Philosophy	239	74	36	42	12	76	208	72	26	42	12	73
Physics & Chemistry	183	66	60	45	11	54	153	78	63	47	15	52
Social Sciences	-	-	-	-	-	-	113	74	33	38	12	58
Others or missing	51	73	35	42	14	9	327	74	46	46	15	35
Total	1441	75	41	43	13	52	2117	79	37	44	14	44

^† ^Professors and other teaching and research staff

- Data not applicable

### Questionnaires

Data on smoking were elicited in the questionnaire by asking whether the respondent currently smoked daily (regularly) or less frequently than every day, or not at all. Those who did not currently smoke were asked if they had previously smoked, and if so on a regular or irregular basis. The questions on smoking were identical in both surveys. The current occupation was asked using response alternatives shown in Additional files 1 and 2. Occupation was re-classified to form higher and lower occupational groups, assigning professors and other teaching and research staff to the higher group and others to the lower group. Education was elicited only in the 2003 survey. Smoking data from national health surveys of the Estonian adult population in 1992 [7] and 2004 [8] were used for reference. The smoking questions used in the national surveys differed in that daily smokers were restricted to those who had smoked that day or the previous day.

### Data analysis

Smoking was classified as daily smoking, ever-smoking (smokes daily or previously did so but has quit) and the quit ratio (previously smoked daily but quit/ever smoked daily, multiplied by 100). Smoking differences between the faculties and other workplaces, and differences between the surveys were examined using a generalized linear model [9] with smoking as a binomial response variate. Since the course of the smoking epidemic is best assessed in terms of absolute changes in prevalence, link function identity was used. The models were fitted by the maximum likelihood method. The explanatory factors were faculty/workplace and survey time, whereas age (as a 3^rd ^degree polynomial) and occupational group (higher/lower) were considered as potential confounding factors. All the analyses were conducted separately for men and women. The size of the population did not allow stratification for any additional factors. The differences between workplaces were expressed in terms of model-based differences (in percentage units) with their 95% confidence intervals (CI) relative to the medical faculty, which was the largest in size. Parameter estimates with their CIs falling above or below the baseline indicate a difference significant at the 5% level, and the width of the CI expresses the precision of the difference estimate. The difference in smoking patterns between the surveys was examined by workplaces, entering the interaction between faculty/workplace and survey time into the model and comparing the prevalence differences predicted by it between the surveys. The consistency of the faculty differences between the surveys and between men and women was examined by regressing the smoking differences in survey II on those observed in survey I, and by similar regressions of women's smoking on men's smoking. These analyses were conducted using ordinary linear regressions weighted by the numbers of respondents.

## Results

### Overall trends

In the first survey, 1343 respondents (97% of those who gave information on faculty/workplace) answered the smoking questions, while in the second survey 1717 (96%) did so. Among the UT employees as a whole, 20% of the men smoked daily in the first survey but only 13% in the second survey. Some decrease was also seen in the crude percentage of ever-smokers, while the quit ratio increased (Table 2). The percentages of daily smokers and ever-smokers were much lower among the UT men than among the general male population of Estonia, and the quit ratios were almost twice as high. The percentages of daily smokers among the UT women were also low compared with the national figures and had declined between the surveys, while the quit ratio had doubled.

**Table 2:** Prevalence of daily smoking, ever smoking and quitting of smoking among the staff at the University of Tartu, Estonia in 1992 (Survey I) and 2003 (Survey II), by faculties and workplaces

	Men				Women			
		
Faculty/workplace	**No**.	Daily (%)	Ever (%)	Quit ratio(%)	**No**.	Daily (%)	Ever (%)	Quit ratio (%)
SURVEY I (1992)								
Biology & Geography	43	30	33	7	31	13	13	0
Administration	23	30	35	13	81	16	19	13
Philosophy	83	25	40	36	146	11	14	24
Medicine	138	24	35	31	189	9	10	11
Economics & Business	27	22	33	33	26	12	15	25
Physics & Chemistry	108	16	32	50	73	5	10	43
Library	16	13	44	71	143	12	16	26
Mathematics & Computing	60	12	37	68	51	6	8	25
Law	27	11	37	70	24	21	21	0
Exercise & Sports Science	25	4	12	67	29	3	10	67
Total	550	20	34	42	793	10	13	21
Estonia, general population 1992^1^		49	65	24		20	24	19

SURVEY II (2003)								
Philosophy	54	24	33	28	149	10	17	40
Library	24	21	46	55	158	8	15	48
Administration	108	19	36	49	139	9	11	20
Medicine	97	14	26	44	252	5	13	59
Biology & Geography	76	11	29	64	95	5	11	50
Education	19	11	21	50	84	6	7	17
Social Sciences	35	9	34	75	74	9	14	30
Mathematics & Computing	32	9	28	67	35	0	3	100
Physics & Chemistry	94	6	16	60	55	7	13	43
Economics & Business	22	5	14	67	42	10	14	33
Exercise & Sports Science	17	0	0	-	28	4	4	0
Law	7	0	0	-	21	10	19	50
Total	585	13	27	53	1132	7	12	43
Estonia, general population 2004^2^		48	71	32		21	40	47

^1 ^From [7]

^            2 ^From [8]

-data not applicable

Where 19% of the men belonging to the higher occupational group and 24% of the lower group smoked daily in the first survey, the trend being similar for the second survey (11% vs 16%), the figures for the women were 8% vs 13% in the first survey and 6% vs 8% in the second.

### Smoking differences between workplaces

#### Men

**Figure 1:**
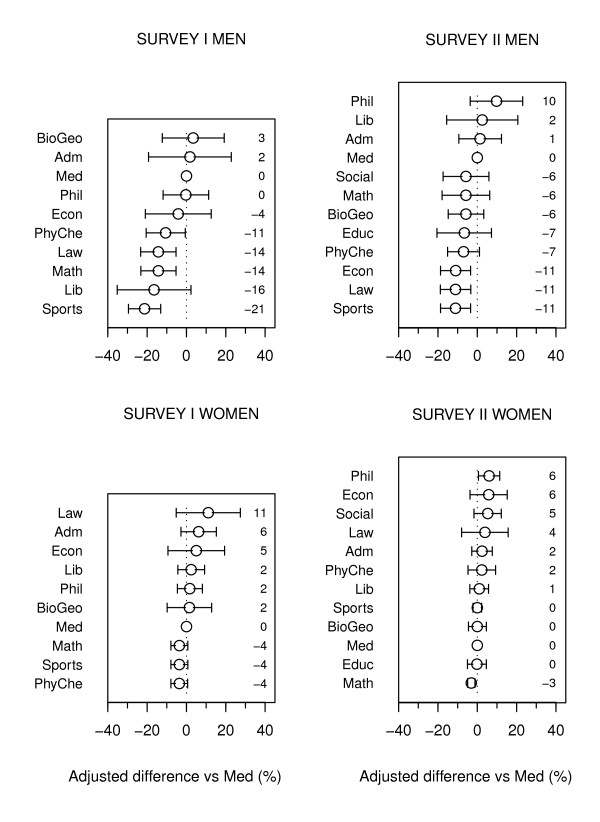
**Smoking differences among University of Tartu employees by faculties and other workplaces**. Circles and numbers show model-based differences vis-à-vis the medical faculty (in percentage points), adjusted for age and occupational group, and horizontal bars indicate their 95% confidence intervals.

The adjusted figures for differences in daily smoking between the medical faculty and the other faculties and workplaces (Figure 1, Additional file 3) varied widely (range 24 and 21 percentage points in the first and second surveys, respectively), being lowest in the faculties of exercise & sports, mathematics, law and physics & chemistry and in the library in the first survey, and still low in most of these, together with the faculty of economics, in the second survey. Most deficits in these low-smoking workplaces vs the medical faculty were significant at the 5% level, the exceptions being the library in the first survey and the faculty of physics & chemistry in the second survey, but the CIs in these workplaces were still more compatible with a deficit than with no deficit compared with the baseline. The men in the faculties of biology & geography and philosophy in the first and second surveys, respectively, were most frequently smokers, although their CIs were quite large and failed to differ significantly from those of the medical faculty.

The men in the medical faculty had relatively high smoking rates in both surveys, so that in no faculty was daily smoking among men significantly more prevalent than in the medical faculty (Figure 1). The quit ratios were mostly higher in non-medical faculties (Table 2, Additional file 3). Despite the wide CIs, the men's quit ratios in the library and in the faculties of law and mathematics in the first survey were significantly higher than those in the medical faculty.

#### Women

Women's daily smoking in the faculties of physics & chemistry, exercise & sports and mathematics was less common than in the medical faculty in the first survey, although despite the narrow CIs, the differences remained below statistical significance at 5%, but only marginally so (Figure 1, Additional file 4). The faculty of law showed the greatest excess over the medical faculty, but this failed to reach statistical significance.

The pattern had become more equivocal by the second survey (Figure 1). Contrary to the men, the women in the medical faculty smoked less than those in most other faculties, although only the faculty of philosophy showed a significantly high figure. The women's quit ratio in the medical faculty actually improved from one of the worst in the first survey to a relatively high one in the second (Table 2, Additional file 4).

### Changes in daily smoking by workplaces

**Figure 2:**
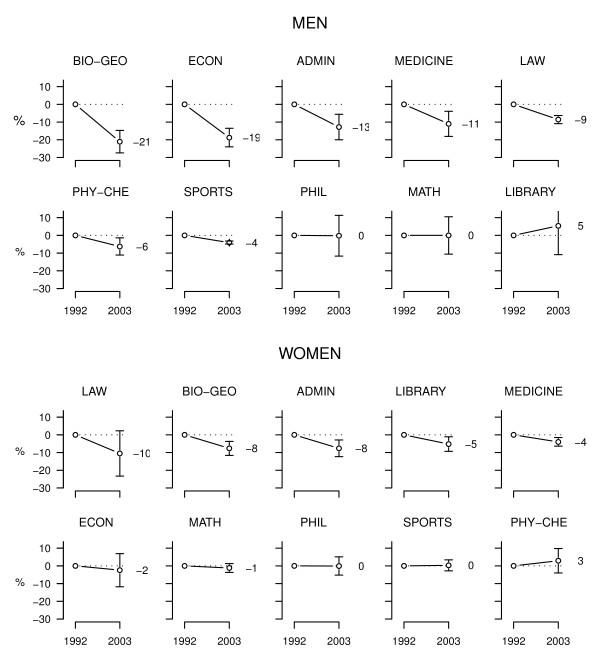
**Estimated change in daily smoking between the surveys among University of Tartu employees, by faculties and other workplaces**. Numbers show model-based changes (in percentage points) between survey I (1992) and survey II (2003), adjusted for age and occupational group, and vertical bars indicate their 95% confidence intervals.

Men's daily smoking decreased significantly in most faculties between the surveys (Figure 2). Smoking decreased most in faculties having high rates initially (e.g. men in the faculties of biology & geography and medicine), with the exception of the faculty of philosophy, where quite many men smoked in the first survey but no decline was seen thereafter. In the faculties having few smokers initially there was little or no change.

Women's smoking declined significantly in only four workplaces (the administrative department, the library, and the faculties of biology & geography and medicine). The decline of 10% in the faculty of law did not quite reach statistical significance, but the CI for the difference between the surveys was still compatible with a decline more than with no decline.

### Consistency of smoking differences

**Figure 3:**
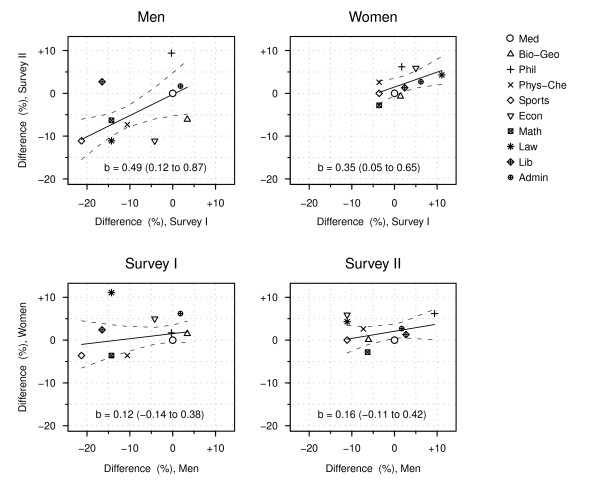
**Consistency of faculty differences in daily smoking between survey I (1992) and survey II (2003), separately for men and women, and between men and women, separately for surveys I and II**. Upper row: Regression of smoking in survey II on smoking in survey I. Lower row: Regression of women's on men's smoking. Points indicate prevalence differences (in percentage points) vis-à-vis the medical faculty, adjusted for age and occupation. Solid lines are weighted linear regressions of the differences and dashed lines their 95% confidence bands. b is the regression slope (95% confidence interval).

The smoking differences between the workplaces in the first survey are compared with those in the second survey in the upper two panels of Figure 3, using adjusted differences in daily smoking vis-à-vis the medical faculty. In both surveys the men in most other faculties smoked less than those in the medical faculty, while the women in most faculties smoked more than those in the medical faculty. The patterns between faculties did not vary independently between the two surveys. While a full correspondence between the surveys would presuppose a regression slope of 1, the observed slope in men was 0.49, indicating that a difference of one percent in men's smoking in the first survey corresponded to a difference of 0.5 per cent in the second survey. The CI of the slope indicates that at a 95% confidence level the slope is not only greater than zero but also less than unity, reflecting the smaller absolute differences (in percentage units) in the second survey. A comparable slope was also seen in women's smoking. Marked deviations from the overall pattern, i.e. workplaces falling outside the 95% confidence band of the linear regression, included men in the faculty of philosophy and in the library, who showed a disproportionately high percentage of smokers in the second survey, while men in the faculties of economics and biology & geography did relatively better in the second survey.

Men's and women's smoking showed almost no association across faculties (the two lower panels in Figure 3). The faculty of law was exceptional in the first survey, since the female employees showed a relatively high figure compared with the males. A similar but much weaker reversal of the sex ratio was also seen in the faculties of law and economics in the second survey.

## Discussion

It was found in the first national health survey conducted in Estonia (1990) that 50% of working-aged men smoked daily and that 40% of men with an academic education did so [10]. We surveyed the UT staff at the same time [5] and found the men's smoking rates to be as low as 20%, although they varied widely from one faculty to another. This led us to the hypothesis that the declining phase of the smoking epidemic among this highly educated community is at a very advanced point, but the course of the epidemic is likely to be affected by differing professional cultures and may perhaps be modified by concurrent changes in society. In this re-survey conducted after Estonia's first transitional decade we show not only that smoking rates among the UT staff have further declined but also that differences still exist between the faculties which are not entirely dissimilar to the previous patterns. The findings can be interpreted in terms of a heterogeneous diffusion of non-smoking behaviour into the most educated segment of the country's population. It remains unclear, however, how well the present findings are applicable to other academic communities.

The validity of our data can be considered to be reasonable. Smoking was elicited using identical questions in the two surveys and the response rates were acceptable, although we cannot rule out biases possibly introduced by selective participation. Thus lower response rates in some faculties, for example, may have led to underestimates of their smoking rates. Since non-responders were not examined, there is a possibility of non-response bias. Smoking is known to be slightly more prevalent among non-respondents [11], but the resulting bias is reportedly small [12]. Underreporting of smoking is a possibility, especially among women [13,14], but it would seem unlikely to have introduced any major flaws. The smoking questions used in the national surveys were slightly more restrictive than the present questions on daily smoking, but the effect of this on smoking rates is considered to be small [8]. Similarly, the lower response rates in the national surveys (63% in both 1992 [7] and 2004 [8]) compared with those in UT can hardly introduce any marked flaw, since the differences in smoking rates were large.

A further limitation is that the smoking differences reported here cannot be interpreted as faculty-specific effects for the university community alone, since 93% of the male respondents and 80% of the females had started their smoking before the age of 25 years, and a half had quit before that age [15]. The results can be understood partly in terms of different mixes of workers entering the faculties or some other kind of selection, and partly as reflecting the effects of the individual's working community. The quit ratios are informative in the latter respect, but as they are not based on an actual follow-up of individual respondents, they are only crude indicators of the propensity to quit. Since we were unable to conduct separate analyses for detailed occupational categories, the results only apply to entire faculties or other workplaces. Differences in occupational composition between the workplaces could have confounded the results, but this factor was allowed for in the analysis.

Even though our sample is larger than those in most other smoking studies conducted among university employees [1,2,16-20], the statistical power was low in some faculties, e.g. the faculty of law. Therefore, the results regarding the smallest strata should be approached with caution. Similar studies performed in other academic communities with pooling of the data would be desirable.

To our knowledge this is the first study to compare smoking patterns between employees in a wide range of university faculties, and especially the first one to assess recent changes in such patterns in a former socialist country. Previous studies have compared smoking between people working in medical and non-medical faculties [1], in combinations of faculties [2], or using only small samples [17,18].

It is frequently observed that members of a medical faculty [1,17-20], doctors in university hospitals [21], and physicians outside academies [22,23] smoke less than employees in other faculties or professions, or people in the general population. Even so, smoking among doctors is regarded as disproportionately common bearing in mind their medical knowledge and their significance as role models [22]. We actually noted one of the highest prevalences of smoking among the men of the medical faculty, although the contrary was true of the women. Only one previous study comparing several faculties has reported a high smoking rate in a medical faculty, but the sample was small and there was no breakdown by sex [16]. In our sample, the relatively low quit ratios among the men in the medical faculty may indicate a poor propensity for behavioural changes or a failure to accept the medical information even though it is readily available. This may not apply to the women in the medical faculty, who showed one of the highest quit ratios in the second survey.

The high prevalence of smoking in the faculty of philosophy (effectively humanities) is a new finding, although students of the humanities and fine arts are said to be smokers [24,25]. The relatively high percentages of daily and ever-smokers in both surveys and the relatively low quit ratios among men and women apparently indicate some life style-related resistance to abstinence from smoking and place this faculty among the last ones to conform to the general decline in smoking.

Another novel finding was the high prevalence of men's smoking in the faculty of biology and geography. Contrary to other faculties having initially high smoking rates, the men in this faculty had greatly reduced their smoking, as also indicated by the increase in the quit ratio from 7% to 64%. The trend is not easily explained, but one might conceive that the growing interest in environmental issues which was a catalyzing factor in the country's independence movement in the late 1980's might have influenced the mix of people entering this faculty and introduced some health-based selection.

Smoking was rare in the faculties of sports & exercise, mathematics and physics & chemistry. Although no similar findings have been reported elsewhere, our findings comply well with the knowledge that physical exercise and smoking are negatively associated [26] and that sportsmen and sportswomen have low smoking rates [27,28]. No such information exists for mathematicians, physicists or chemists, however, except that it has been conjectured that students of mathematics may have low smoking rates [25]. We assume that the harmful effects of smoking on physical and cognitive performance [29-31], perhaps also reflected in poorer school results [32,33] and educational achievements [34] among smokers, have prevented people in these faculties from smoking.

The faculty of law was the only one where women smoked more than men in both surveys, and a difference of a similar kind was seen in the faculty of economics in the second survey. These faculties have had the closest connection with the recent changes in Estonian society, and the reversed sex ratio probably indicates the women's intensified drive for equality, and perhaps also coping-related stress under rapidly changing conditions. No other study has analysed sex ratios in smoking among an academic population in a transitional society.

## Conclusions

Smoking differences at a given point in time are best interpreted as a snapshot from a longitudinal process, the smoking epidemic [6]. The declining phase of the epidemic has started only recently in Estonia as a whole [35], but it may have started some decades ago within the country's academic community [36]. We have shown here that even within this highly educated segment of the population a non-smoking form of behaviour is adopted in a heterogeneous manner, and paradoxically, not necessarily in the medical faculty first, but rather in faculties where physical or mental performance is of particular significance. We assume that the smoking epidemic in UT is now approaching its terminal phase, some of its faculties being almost smoke-free, which is in sharp contrast to the persistently high smoking rates in the population as a whole. Local anti-smoking actions are still needed and should be directed at male employees in the faculty of philosophy, which now lags behind, and also at men in the medical faculty, which should lead the way to a smoke-free academic community. As UT is the intellectual and cultural hub of Estonia, it serves as a major centre of innovation [6], and successful efforts within this community could speed up the diffusion of non-smoking throughout the country.

## Competing interests

The authors declare that they have no competing interests.

## Authors' contributions

The study was conceived by PHK, RH and JK. The data analysis was performed by SN and RH, and the manuscript was first drafted by RH and revised and finalized by SN, JK and PHK. All the authors have read and approved the manuscript. PHK and SN are the guarantors of the study.

## Pre-publication history

The pre-publication history for this paper can be accessed here:

http://www.biomedcentral.com/1471-2458/11/153/prepub

## Supplementary Material

Additional file 1 **Survey questionnaire in 1992**. The survey questionnaire used to obtain data in 1992 translated from Estonian into English. Click here for file

Additional file 2 **Survey questionnaire in 2003**. The survey questionnaire used to obtain data in 2003 translated from Estonian into English. Click here for file

Additional file 3 **Smoking differences among University of Tartu male employees by faculties and other workplaces**. Differences in daily smoking, ever-smoking and quit ratios among University of Tartu male employees by faculties and other workplaces vis-à-vis the medical faculty, adjusted for age and occupational group. Click here for file

Additional file 4 **Smoking differences among University of Tartu female employees by faculties and other workplaces**. Differences in daily smoking, ever-smoking and quit ratios among University of Tartu female employees by faculties and other workplaces vis-à-vis the medical faculty, adjusted for age and occupational group. Click here for file
