# Supplementary material for: Smoking differences between employees in faculties of the University of Tartu, Estonia, and changes during the country's transition
Source: BMC Public Health. 2011 Mar 8;11:153. doi: 10.1186/1471-2458-11-153 (PMC3065408; doi:10.1186/1471-2458-11-153)
Supplement: Additional file 1 — Survey questionnaire in 1992. The survey questionnaire used to obtain data in 1992 translated from Estonian into English. [file 1471-2458-11-153-S1.DOC]

| SMOKING STUDY AMONG EMPLOYEES OF THE UNIVERSITY OF TARTU | |
| --- | --- |
| The employees of the University of Tartu have not recently been surveyed for their smoking habits. Since respiratory diseases are on the increase, it is necessary to investigate the reasons for this. Smoking is a risk factor, not only for respiratory diseases, but also for diseases of the circulatory organs. The purpose of this questionnaire is therefore to obtain information on smoking habits.  This questionnaire has been drawn up by Leo Hirvonen, a professor of physiology at the University of Oulu, and the present inquiry is part of the international cooperation between the universities of Tartu and Oulu. This inquiry is being conducted with the permission of the administration of the University of Tartu. The information of the questionnaire will be collected from all of the employees of the University of Tartu who were mentioned in the most up-to-date staff records.  - Please read the whole questionnaire through, regardless of whether you smoke or not.  - The alternatives for responses are marked by numbers. Please draw a circle around the appropriate number.  - If you do not smoke, please answer questions 1-12.  - If you do smoke, please answer all the questions. | |
| | 1. Gender  Male………………………………………….……… 1  Female………………………………………………. 2 | | --- | | 2. Year of birth…………………………………19____ | | 3. Marital status  Single……………………………………….……… 1  Cohabiting 2  Married 3  Separated 4  Divorced 5  Widow/Widower 6 | | 4. Worksite at the university  Administrative department 1  Faculty of medicine 2  Faculty of biology and geography 3  Faculty of philosophy 4  Faculty of physics and chemistry 5  Faculty of sports and exercise 6  Faculty of economics 7  Faculty of mathematics 8  Faculty of theology. 9  Faculty of law 10  Library 11  Other (please specify) 12 | |  | | | 5. Current occupation  Professor 1  Senior teacher 2  Teaching assistant 3  Senior researcher 4  Researcher 5  Laboratory worker 6  Engineer 7  Technician 8  Secretary 9  Auxiliary personnel 10  Laboratory assistant 11  Office employee 12  Other position 13 | | --- | | 6. Do you currently smoke?  I smoke daily (regularly) 1  I smoke less frequently than every day 2  I do not smoke at all 3 | | 7. If you do not currently smoke, have you previously smoked?  I have smoked regularly 1  I have smoked irregularly 2  I have only tried smoking 3  I have never tried smoking 4 | |

| |  | | --- | | 8. If you have smoked at some time, at what age did you smoke for the first time?  At the age of……..……………................_______ years. | |  | | 9. If you have smoked regularly, at what age did you start smoking regularly?  At the age of……..……………................_______ years. | |  | | 10. If you have smoked previously, but do not currently smoke,  A) Why did you quit?  Disease 1  Deteriorated health 2  Economic reasons 3  Reasons of hygiene 4  Working conditions 5  Family reasons 6  The influence of friends 7  Health education 8  Ethical reasons 9  Other reasons 10 | | B) When did you give up smoking?  During the past six months 1  6 months-2 years ago 2  More than 2 years ago 3  More than 10 years ago 4 | | 11. What is your opinion about the health effects of smoking?  No significant effect 1  Harmful effect 2  Beneficial effect 3  Both harmful and beneficial effects 4  I cannot say 5 | | 12. What is you personal experience about the health effects of smoking?  No personal experience 1  No effect worth mentioning 2  Harmful effect 3  Beneficial effect 4  Both harmful and beneficial effects 5 | | 13. If you smoke, what kind of tobacco do you smoke and how much? If you use more than one kind, please answer all of the response alternatives.  Non-filter cigarettes ___ no/day 1  Filter cigarettes ___ no/day 2  Paper mouthpiece cigarettes ___ no/day 3  Oriental cigarettes ___ no/day 4  Shag tobacco in a pipe ___ g/day 5  Shag tobacco in cigarettes ___ g/day 6  Cigars ___ no/day 7  Cigarillos ___ no/day 8  Snuff ___ g/day 9  Chewing tobacco ___ g/day 10  Total amount per day, _____  or grams per week _____ | | | 14. Do you inhale the smoke into your lungs?  Usually…………………………………………….. 1  Mostly…………………………………………….. 2  Seldom……………………………………………… 3  Never………………………………………………. 4 | | --- | | 15. Have you ever tried to give up smoking?  Never 1  Over the past six months 2  Some time previously 3 | | 16. Would you seriously want to give up smoking?  No 1  Yes, I would on my own 2  Yes, in a small group 3  Yes, in a withdrawal group or courses 4 | | 17. Do you think that you would be successful in quitting?  I can give up any time I want 1  I would easily be successful 2  I believe that I would be successful despite difficulties 3  I am afraid of failure 4  I do not believe that I would be successful 5 | | 18. Why do you want to give up smoking?  Health reasons 1  Economic reasons 2  Ethical and aesthetic reasons 3  Other reasons 4  I cannot say 5 | | 19. Give reasons for why you want to continue smoking?  I like it and it is enjoyable 1  I do not regard it to be harmful 2  As a habit 3  It helps me during mental work and studying 4  It eases my personal relationships, for company 5  It calms me 6  I stimulates me 7  I am addicted to smoking 8  Other reasons 9  I cannot say 10 | |  | |
| --- | --- | --- | --- | --- | --- | --- | --- | --- | --- | --- | --- | --- | --- | --- | --- | --- | --- | --- |
