# Supplementary material for: Smoking differences between employees in faculties of the University of Tartu, Estonia, and changes during the country's transition
Source: BMC Public Health. 2011 Mar 8;11:153. doi: 10.1186/1471-2458-11-153 (PMC3065408; doi:10.1186/1471-2458-11-153)
Supplement: Additional file 2 — Survey questionnaire in 2003. The survey questionnaire used to obtain data in 2003 translated from Estonian into English. [file 1471-2458-11-153-S2.DOC]

UNIVERSITY OF TARTU

Department of Physiology

Dear member of the Tartu University staff !

The questionnaire at hand continues the smoking studies of university employees, conducted in cooperation with the universities of Tartu and Oulu. The study is concurrently being carried out in both universities. Smoking habits among the employees of the University of Tartu are now being surveyed for the second time. Such a comparative study, arranged by universities of two neighbouring countries, was conducted for the first time in 1991 in the University of Oulu and through 1992-93 in the University of Tartu. The information which was collected more than 10 years ago showed that among the University of Oulu staff, 13% of men and 10% of women smoked, while the respective figures in the University Tartu were 21% and 12%

The present inquiry will help us to clarify what the situation is now and what kinds of changes have occurred in one of the most important risk factors concerning health among university employees, after Estonia regained its independence.

Please answer the questions carefully, place the filled questionnaire into the returning envelope and send it via internal mail to the address mentioned on the envelope.

All of the information obtained by the questionnaire will be treated anonymously and used for scientific purposes only. The number marked in the questionnaire is needed to identify those who have not replied to the questionnaire and to whom we can send a reminder.

The study is funded by the University of Oulu.

We are very grateful for your answers and your participation in the study.

Peet-Henn Kingisepp

Professor, emeritus

Department of Physiology, University of Tartu

| SMOKING STUDY AMONG EMPLOYEES OF THE UNIVERSITY OF TARTU | |
| --- | --- |
| Please read the whole questionnaire through and answer each question regardless of whether you smoke or not. In each question, please circle the correct alternative (some questions may have more than one), or write a number or an answer in an appropriate place. Your answers are very important, regardless of whether you smoke daily, occasionally or not at all. We are very grateful for your answers! | |
| | 1. Gender  Male………………………………………….……… 1  Female………………………………………………. 2 | | --- | | 2. Year of birth…………………………………19____ | | 3. Marital status  Single……………………………………….……… 1  Cohabiting 2  Married 3  Separated 4  Divorced 5  Widow/Widower 6 | | 4. Worksite at the university  Administrative department 1  Faculty of philosophy 2  Faculty of education 3  Faculty of biology and geography 4  Faculty of medicine 5  Faculty of economics 6  Faculty of physics and chemistry 7  Faculty of sports and exercise 8  Faculty of mathematics and information technology. 9  Faculty of theology 10  Faculty of law 11  Faculty of social sciences 12  Library 13  Other (please specify) 14 | | 5. Current occupation  Professor or other teaching staff/researcher 1  Laboratory chief, engineer or other laboratory  employee 2  Employee in information technology, publication or  other services 3  Clerical or administrative employee 4  Other position 5 | | 6. Your education  Doctor 1  Master 2  Bachelor 3  Polytechnic 4  College 5  Vocational school 6  Matriculation 7  Without matriculation 8 | | 7. Do you or another family member  smoke at home?  No one smokes 1  Yes, I smoke 2  Yes, someone else in my family smokes 3 | | | 8. How many hours a day do you work in a smoky location at your workplace?  More than 5 hours 1  1-5 hours 2  Less than one hour a day 3  None at all 4 | | --- | | 9. How is smoking arranged at your workplace?  No one smokes anywhere 1  Smoking is allowed outdoors 2  Smoking indoors is only allowed in smoking rooms 3  Smoking indoors is allowed in both smoking rooms  and in some working rooms 4  Smoking is also allowed in other indoor spaces 5 | | 10. Are you satisfied with the smoking arrangements at  your workplace?  No 1  Yes 2 | | 11. Do you currently smoke?  I smoke daily (regularly) 1  I smoke less frequently than every day 2  I do not smoke at all 3 | | 12. If you do not currently smoke, have you previously smoked?  I have smoked regularly 1  I have smoked irregularly 2  I have only tried smoking 3  I have not even tried smoking 4 | | 13. At what age did you try smoking for the first time?  (Enter 99, if you have never tried.)  At the age of………………..………........____ years. | | 14. At what age would you have smoked 100 times (cigarettes, cigarillos, pipe or snuff)? (Enter 99, if you  have never smoked)  At the age of ……………..…..…………..____ years | | 15. At what age did you start smoking regularly?  (Enter 99, if you have never smoked.)  At the age of …………..……………........____ years | | 16. If you have previously smoked, but do not currently smoke,  A) Why did you quit? (you can give several reasons)  Disease 1  Deteriorated health 2  Economic reasons 3  Reasons of hygiene 4  Working conditions 5  Family reasons 6  The influence of friends 7  Health education 8  Ethical reasons 9  Other reasons 10 | |

| | B) When did you give up smoking?  During the past six months 1  6 months-2 years ago 2  More than 2 years ago 3  More than 10 years ago 4 | | --- | | C) At what age did you give up smoking…..____ years  17. What is your opinion about the health effects of smoking?  No significant effect 1  Harmful effect 2  Beneficial effect 3  Both harmful and beneficial effects 4  I cannot say 5 | | 18. What is you personal experience about the health effects of smoking?  No personal experience 1  No effect worth mentioning 2  Harmful effect 3  Beneficial effect 4  Both harmful and beneficial effects 5 | | If you have never smoked, you can stop here. Thank you for your answers!  If you currently smoke or have previously smoked but have quit, please continue. | | 19. What kind of tobacco and how much do you currently consume (or consumed before quitting, if you do not currently smoke)? Regarding the various kinds of tobacco, please carefully mark, for each kind, the amount consumed as carefully as you can, either as numbers (e.g. cigarettes) or grams (g).   |  | Currently | Previously |  | | --- | --- | --- | --- | | Non-filter cigarettes | 1___ | 1___ | no/day | | Filter cigarettes | 2___ | 2___ | no/day | | Paper mouthpiece cigarettes | 3___ | 3___ | no/day | | Oriental cigarettes | 4___ | 4___ | no/day | | Shag tobacco in a pipe | 5___ | 5___ | g/day | | Shag tobacco in cigarettes | 6___ | 6___ | g/day | | Cigars | 7___ | 7___ | no/day | | Cigarillos | 8___ | 8___ | no/day | | Snuff | 9___ | 9___ | g/day | | Chewing tobacco | 10___ | 10___ | g/day | | Total amount, either | _____ | _____ | no/day | | or | _____ | _____ | g/day | | | 20. Do you inhale the smoke into your lungs (or did you inhale before quitting, if you do not smoke currently)?  Usually…………………………………………….. 1  Mostly…………………………………………….. 2  Seldom……………………………………………… 3  Never………………………………………………. 4 | | 21. How much time normally passed between waking up in the morning and smoking your first tobacco (or did so before you quit)?  During the first five minutes………………………… 1  During 5-30 minutes…………………………………. 2  During 31-60 minutes 3  After the first hour…………………………………… 4 | | | 22. Have you ever used bupropione (Zyban) in order to give up smoking?  No 1  I have used it previously 2  I currently use it 3 | | --- | | 23. Have you ever used nicotine replacement therapy  (such as chewing gum, patches, tablets, spray, etc)?  No 1  Yes, during the past 12 months. 2  Yes, some time previously 3  I currently use them 4 | | If you do not smoke any more, you can stop here. Thank you for your answers! If you currently smoke, please also answer the remainder of the questions. | | 24. Have you ever tried to give up smoking altogether?  Never 1  Over the past six months 2  Some time previously 3 | | 25. Would you seriously want to give up smoking?  No 1  Yes, I would on my own 2  Yes, in a small group 3  Yes, in a withdrawal group or course 4  Otherwise, how________________________________5 | | 26. Do you think that you would be successful in quitting?  I can give up any time I want 1  I would easily be successful 2  I believe that I would be successful despite difficulties 3  I am afraid of failure 4  I do not believe that I would be successful 5 | | 27. For what reasons (there may be more than one) would  you like to give up smoking?  Health reasons 1  Economic reasons 2  Ethical and aesthetic reasons 3  Other reasons 4  I cannot say 5 | | 28. For what reasons (there may be more than one) would  you like to continue smoking?  I like it and it is enjoyable 1  I do not regard it to be harmful 2  As a habit 3  It helps me during mental work and studying 4  It eases my personal relationships, for company 5  It calms me 6  I stimulates me 7  I am addicted to smoking 8  Other reasons 9  I cannot say 10 | |
| --- | --- | --- | --- | --- | --- | --- | --- | --- | --- | --- | --- | --- | --- | --- | --- | --- | --- | --- | --- | --- | --- | --- | --- | --- | --- | --- | --- | --- | --- | --- | --- | --- | --- | --- | --- | --- | --- | --- | --- | --- | --- | --- | --- | --- | --- | --- | --- | --- | --- | --- | --- | --- | --- | --- | --- | --- | --- | --- | --- | --- | --- | --- | --- | --- | --- | --- | --- | --- |
