# Supplementary material for: Smoking differences between employees in faculties of the University of Tartu, Estonia, and changes during the country's transition
Source: BMC Public Health. 2011 Mar 8;11:153. doi: 10.1186/1471-2458-11-153 (PMC3065408; doi:10.1186/1471-2458-11-153)
Supplement: Additional file 3 — Smoking differences among University of Tartu male employees by faculties and other workplaces. Differences in daily smoking, ever-smoking and quit ratios among University of Tartu male employees by faculties and other workplaces vis-à-vis the medical faculty, adjusted for age and occupational group. [file 1471-2458-11-153-S3.DOC]

| **Additional file 3**  Smoking differences among University of Tartu male employees by faculties and other workplaces. Figures are model-based differences vis-à-vis the medical faculty (in percentage points), adjusted for age and occupational group. 95% confidence intervals of the differences are shown in parentheses. | | | | | |
| --- | --- | --- | --- | --- | --- |
| Survey I (1992) | | |  | Survey II (2003) | |
| Faculty/workplace | Difference | |  | Faculty/workplace | Difference |
| DAILY SMOKING | | | | | |
| Biology & Geography | | 3 (-12 to 19) |  | Philosophy | 10 (-4 to 23) |
| Administration | | 2 (-19 to 23) |  | Library | 2 (-16 to 21) |
| Medicine (reference) | | 0 |  | Administration | 1 (-9 to 12) |
| Philosophy | | 0 (-12 to 11) |  | Medicine (reference) | 0 |
| Economics & Business | | -4 (-21 to 13) |  | Social Sciences | -6 (-17 to 6) |
| Physics & Chemistry | | -11 (-21 to -1) |  | Mathematics & Computing | -6 (-18 to 6) |
| Law | | -14 (-23 to -5) |  | Biology & Geography | -6 (-15 to 3) |
| Mathematics & Computing | | -14 (-23 to -5) |  | Education | -7 (-21 to 7) |
| Library | | -16 (-35 to 2) |  | Physics & Chemistry | -7 (-15 to 1) |
| Exercise & Sports Science | | -21 (-30 to -13) |  | Economics & Business | -11 (-19 to -3) |
|  | |  |  | Law | -11 (-19 to -3) |
|  | |  |  | Exercise & Sports Science | -11 (-19 to -3) |
| EVER SMOKED DAILY | | | | | |
| Library | | 5 (-22 to 32) |  | Library | 17 (-5 to 40) |
| Philosophy | | 4 (-9 to 17) |  | Administration | 9 (-4 to 22) |
| Law | | 2 (-18 to 22) |  | Social Science | 8 (-10 to 26) |
| Medicine (reference) | | 0 |  | Philosophy | 5 (-10 to 21) |
| Mathematics & Computing | | 0 (-15 to 14) |  | Biology & Geography | 3 (-10 to 17) |
| Economics & Business | | -2 (-22 to 17) |  | Mathematics & Computing | 1 (-17 to 19) |
| Biology & Geography | | -3 (-19 to 14) |  | Medicine (reference) | 0 |
| Administration | | -6 (-28 to 16) |  | Education | -7 (-26 to 13) |
| Physics & Chemistry | | -6 (-18 to 6) |  | Physics & Chemistry | -10 (-21 to 2) |
| Exercise & Sports Science | | -18 (-31 to -5) |  | Economics & Business | -16 (-27 to -4) |
|  | |  |  | Exercise & Sports Science | -16 (-28 to -5) |
|  | |  |  | Law | -20 (-30 to -10) |
| QUIT RATIO | | | | | |
| Library | | 58 (18 to 99) |  | Social Science | 34 (-9 to 59) |
| Law | | 39 (16 to 61) |  | Library | 29 (-8 to 65) |
| Mathematics & Computing | | 38 (14 to 61) |  | Economics & Business | 26 (-32 to 82) |
| Exercise & Sports Science | | 23 (-45 to 90) |  | Mathematics & Computing | 22 (-15 to 59) |
| Physics & Chemistry | | 19 (-2 to 40) |  | Biology & Geography | 21 (-4 to 46) |
| Philosophy | | 7 (-12 to 27) |  | Physics & Chemistry | 19 (-10 to 48) |
| Economics & Business | | 5 (-27 to 36) |  | Administration | 12 (-14 to 38) |
| Medicine (reference) | | 0 |  | Education | 6 (-46 to 57) |
| Biology & Geography | | -12 (-26 to 1) |  | Law | - |
| Administration | | -15 (-31 to 1) |  | Exercise & Sports Science | - |
|  | |  |  | Medicine | 0 |
|  | |  |  | Philosophy | -14 (-44 to 15) |
| - not applicable (no respondent had ever smoked) | | | | | |
