# Supplementary material for: Smoking differences between employees in faculties of the University of Tartu, Estonia, and changes during the country's transition
Source: BMC Public Health. 2011 Mar 8;11:153. doi: 10.1186/1471-2458-11-153 (PMC3065408; doi:10.1186/1471-2458-11-153)
Supplement: Additional file 4 — Smoking differences among University of Tartu female employees by faculties and other workplaces. Differences in daily smoking, ever-smoking and quit ratios among University of Tartu female employees by faculties and other workplaces vis-à-vis the medical faculty, adjusted for age and occupational group. [file 1471-2458-11-153-S4.DOC]

| **Additional file 4** Smoking differences among University of Tartu female employees by faculties and other workplaces. Figures are model-based differences vis-à-vis the medical faculty (in percentage points), adjusted for age and occupational group. 95% confidence intervals of the differences are shown in parentheses. | | | | | |
| --- | --- | --- | --- | --- | --- |
| Survey I (1992) | |  | Survey II (2003) | | |
| Faculty/workplace | Difference |  | Faculty/workplace | | Difference |
| DAILY SMOKING | | | | | |
| Law | 11 (-5 to 27) |  | Philosophy | 6 (1 to 11) | |
| Administration | 6 (-3 to 15) |  | Economics & Business | 6 (-4 to 15) | |
| Economics & Business | 5 (-9 to 19) |  | Social Sciences | 5 (-2 to 12) | |
| Library | 2 (-4 to 9) |  | Law | 4 (-8 to 16) | |
| Philosophy | 2 (-5 to 8) |  | Administration | 2 (-3 to 8) | |
| Biology & Geography | 2 (-10 to 13) |  | Physics & Chemistry | 2 (-5 to 9) | |
| Medicine (reference) | 0 |  | Library | 1 (-4 to 6) | |
| Mathematics & Computing | -4 (-8 to 1) |  | Exercise & Sports Science | 0 (-2 to 2) | |
| Exercise & Sports Science | -4 (-8 to 1) |  | Biology & Geography | 0 (-5 to 5) | |
| Physics & Chemistry | -4 (-8 to 1) |  | Medicine | 0 | |
|  |  |  | Education | 0 (-5 to 5) | |
|  |  |  | Mathematics & Computing | -3 (-5 to -1) | |
| EVER SMOKED DAILY | | | | | |
| Law | 9 (-8 to 25) |  | Law | 9 (-9 to 26) | |
| Administration | 7 (-3 to 17) |  | Philosophy | 6 (-1 to 13) | |
| Economics & Business | 6 (-10 to 22) |  | Economics & Business | 4 (-7 to 16) | |
| Library | 4 (-4 to 12) |  | Social Sciences | 3 (-5 to 12) | |
| Philosophy | 3 (-4 to 10) |  | Physics & Chemistry | 2 (-8 to 12) | |
| Exercise & Sports Science | 1 (-11 to 13) |  | Library | 1 (-6 to 8) | |
| Medicine (reference) | 0 |  | Medicine | 0 | |
| Biology & Geography | 0 (-11 to 11) |  | Biology & Geography | -1 (-8 to 6) | |
| Physics & Chemistry | -3 (-10 to 4) |  | Administration | -3 (-10 to 3) | |
| Mathematics & Computing | -5 (-11 to 1) |  | Exercise & Sports Science | -3 (-10 to 3) | |
|  |  |  | Education | -6 (-12 to 0) | |
|  |  |  | Mathematics & Computing | -8 (-13 to -3) | |
| QUIT RATIO | | | | | |
| Exercise & Sports Science | 54 (3 to 100) |  | Mathematics & Computing | 40 (17 to 62) | |
| Physics & Chemistry | 38 (1 to 74) |  | Social Sciences | 34 (9 to 59) | |
| Library | 19 (1 to 37) |  | Education | 6 (-46 to 57) | |
| Mathematics & Computing | 18 (-22 to 59) |  | Medicine (reference) | 0 | |
| Economics & Business | 16 (-27 to 59) |  | Biology & Geography | -10 (-45 to 26) | |
| Philosophy | 12 (-6 to 31) |  | Library | -12 (-40 to 17) | |
| Administration | 0 (-1 to 1) |  | Law | -16 (-67 to 37) | |
| Law | 0 (- 2 to 2) |  | Physics & Chemistry | -19 (-60 to 21) | |
| Biology & Geography | 0 (-1 to 1) |  | Philosophy | -29 (-55 to -3) | |
| Medicine (reference) | 0 |  | Economics & Business | -31 (-72 to 10) | |
|  |  |  | Administration | -39 (-67 to -11) | |
|  |  |  | Exercise & Sports Science | -57 (-78 to -36) | |
